# First and second trimester urinary metabolic profiles and fetal growth restriction: an exploratory nested case-control study within the infant development and environment study

**DOI:** 10.1186/s12884-018-1674-8

**Published:** 2018-02-08

**Authors:** Gauri Luthra, Ivan Vuckovic, A. Bangdiwala, H. Gray, J. B. Redmon, E. S. Barrett, S. Sathyanarayana, R. H. N. Nguyen, S. H. Swan, S. Zhang, P. Dzeja, S. I. Macura, K. S. Nair

**Affiliations:** 1 Nuclear Magnetic Resonance Facility, Mayo Clinic, Stabile SL-035, 200 First Street SW, Rochester, MN 55905 USA; 2 Clinical and Translational Science Institute, University of Minnesota, 717 Delaware Street SE, Second Floor, Minneapolis, MN 55414 USA; 3 Department of Maternal Fetal Medicine, University of Minnesota, 606 24th Ave S #400, Minneapolis, MN 55454 USA; 4 Division of Diabetes Endocrinology and Metabolism, 516 Delaware Street SE, MMC 101, Minneapolis, MN 55455 USA; 5 Environmental and Occupational Health Sciences Institute, Rutgers School of Public Health, 170 Frelinghuysen Rd, Piscataway, NJ 08854 USA; 6 Department of Pediatrics, University of Washington Seattle Children’s Research Institute, CW8-6, PO Box 5371, Seattle, WA 98145-5005 USA; 7 Division of Epidemiology and Community Health, University of Minnesota, 1300 S. 2nd Street, Suite 300, Minneapolis, MN 55454 USA; 8 Department of Preventive Medicine, Icahn School of Medicine at Mount Sinai, 1 Gustave L. Levy Pl, New York, NY 10029 USA; 9 Metabolomics Core, Mayo Clinic Hospital, Saint Mary’s Campus, Alfred Building, Fifth Floor, Room 417, 200 First St. SW, Rochester, MN 55905 USA

**Keywords:** Obstetrics, NMR spectroscopy, Fetal growth restriction

## Abstract

**Background:**

Routine prenatal care fails to identify a large proportion of women at risk of fetal growth restriction (FGR). Metabolomics, the comprehensive analysis of low molecular weight molecules (metabolites) in biological samples, can provide new and earlier biomarkers of prenatal health. Recent research has suggested possible predictive first trimester urine metabolites correlating to fetal growth restriction in the third trimester. Our objective in this current study was to examine urinary metabolic profiles in the first and second trimester of pregnancy in relation to third trimester FGR in a US population from a large, multi-center cohort study of healthy pregnant women.

**Methods:**

We conducted a nested case-control study within The Infant Development and the Environment Study (TIDES), a population-based multi-center pregnancy cohort study. We identified 53 cases of FGR based on the AUDIPOG [Neonatal growth - AUDIPOG [Internet]. [cited 29 Nov 2016]. Available from: http://www.audipog.net/courbes_morpho.php?langue=en] formula for birthweight percentile considering maternal height, age, and prenatal weight, as well as infant sex, gestational age, and birth rank. Cases were matched to 106 controls based on study site, maternal age (± 2 years), parity, and infant sex. NMR spectroscopy was used to assess concentrations of four urinary metabolites that have been previously associated with FGR (tyrosine, acetate, formate, and trimethylamine) in first and second trimester urine samples. We fit multivariate conditional logistic regression models to estimate the odds of FGR in relation to urinary concentrations of these individual metabolites in the first and second trimesters. Exploratory analyses of custom binned spectroscopy results were run to consider other potentially related metabolites.

**Results:**

We found no significant association between the relative concentrations of each of the four metabolites and odds of FGR. Exploratory analyses did not reveal any significant differences in urinary metabolic profiles. Compared with controls, cases delivered earlier (38.6 vs 39.8, *p* < 0.001), and had lower birthweights (2527 g vs 3471 g, *p* < 0.001). Maternal BMI was similar between cases and controls.

**Conclusions:**

First and second trimester concentrations of urinary metabolites (acetate, formate, trimethylamine and tyrosine) did not predict FGR. This inconsistency with previous studies highlights the need for more rigorous investigation and data collection in this area before metabolomics can be clinically applied to obstetrics.

**Electronic supplementary material:**

The online version of this article (10.1186/s12884-018-1674-8) contains supplementary material, which is available to authorized users.

## Background

Fetal growth restriction (FGR) is a complication of pregnancy that has been associated with a variety of adverse perinatal outcomes including intrauterine fetal demise, neonatal morbidity, and neonatal death. Studies have revealed that growth-restricted fetuses are predisposed to the development of cognitive delay in childhood and diseases in adulthood such as obesity, type 2 diabetes mellitus, coronary artery disease, and stroke. [1] The FGR incidence is reported to be approximately 4% to 8% in developed countries, including the United States, and 6% to 30% in developing countries. [2] Routine prenatal care fails to identify a large proportion of women at risk; therefore, there is an imperative need to identify the risk of FGR early in pregnancy so that it might be prevented.

FGR is commonly defined as an estimated fetal weight that is less than the 10th percentile for gestational age. [3] In the research literature, FGR is often mistakenly interchanged with ‘small for gestational age’ (SGA, birthweight below the 10th percentile for the gestational age) [4] or ‘low birthweight’ (defined as birth weight less than 2500 g) [5], to describe the same phenomenon; however, these terms are not necessarily synonymous. It has been estimated that approximately one third of all SGA infants are growth restricted (hence two thirds are constitutionally small) [2]; additionally, not all growth restricted babies are SGA. For our study, we defined FGR as failure to achieve the individual baby’s growth potential as defined by the specific growth potential formula from AUDIPOG. [6] Mamelle et al. [7] illustrates the benefits of using this formula along with a discussion clarifying the concepts of SGA and FGR and their relationships with one another.

In a 2014 article by Maitre et al. [8], the authors found a significant correlation between lower levels of four urinary metabolites (acetate, formate, tyrosine and trimethylamine) and a higher incidence of FGR by performing NMR spectroscopy on first trimester urinary samples of pregnant patients in a cohort from Greece. They applied the AUDIPOG growth potential formula to better identify growth restricted fetuses. The exact role that these four metabolites play in relation to possible pathology causing FGR is yet to be discovered though they have been associated with nutrition and colonic health and tyrosine associated with phenylketonuria. [9–11]

Metabolomics, the comprehensive analysis of low molecular weight molecules (metabolites) in biological samples, can provide new and earlier biomarkers of prenatal health. [12–15] Studies also have shown a possible link between metabolites and the intrauterine environment and the effect it can have on fetal development and physiological systems during gestation as well as later in life. [16, 17] As we learn more about the intrauterine environment and how it is influenced by metabolites, we hope to find an early, non-invasive way to identify pregnancies at risk of FGR.

Our objective in this current study was to examine urinary metabolic profiles in the first and second trimester of pregnancy in relation to third trimester FGR in a US population from a large, multi-center cohort study of healthy pregnant women.

## Methods

We used data from The Infant Development and the Environment Study (TIDES) [18, 19] in which, from 2010 to 2012, pregnant women were recruited from obstetrical clinics affiliated with academic medical centers in four U.S. cities: Minneapolis, MN; Rochester, NY; San Francisco, CA; and Seattle, WA. The primary aim of the TIDES study was to examine prenatal phthalate exposure in relation to infant genital morphology. Participants gave a urine sample in each trimester, which was collected at a study visit and frozen. Participants also completed a questionnaire in each trimester regarding demographics and possible environmental exposures.

For our case-control study, we reviewed these questionnaires and de-identified previously collected data that included sociodemographic variables such as maternal age, maternal education, and woman’s parity, along with known contributing factors to FGR, including smoking status of the mother during pregnancy, chronic hypertension, and pre-gestational diabetes. Though pre-gestational diabetes is most commonly associated with fetal overgrowth (macrosomia), there is a subset of patients where impaired growth is more common among women with diabetic vasculopathy due to impaired placental function as well as fetal demise. [20] Per The American College of Obstetricians and Gynecologists (ACOG), for patients with pre-gestational diabetes in the setting of hypertension and nephropathy, the risk of fetal intrauterine growth restriction is more than doubled. [21]

### Identification of FGR cases and controls

FGR cases were determined using the AUDIPOG formula for the average predicted birthweight for an infant with specific characteristics of: maternal height, age, and prenatal weight, as well as infant sex, gestational age, and birth rank.

AUDIPOG formula [6]:

$$ {\displaystyle \begin{array}{l}\mathrm{avg}\_\mathrm{pred}\_\mathrm{bw}=10,228066774-0,646727171\ast \mathrm{GA}+0,0259713417\ast {\mathrm{GA}}^2\\ {}-0,000291122\ast {\mathrm{GA}}^3-0,045467351\ast \mathrm{sex}+0,0606013862\ast \operatorname{rank}\\ {}-0,013592585\ast {\operatorname{rank}}^2+0,0009109473\ast {\operatorname{rank}}^3+0,0003976103\ast \mathrm{MA}\\ {}+0,0019992269\ast \mathrm{MH}+0,0169049061\ast \mathrm{MW}-0,000171266\ast {\mathrm{MW}}^2\\ {}+5,8340462\mathrm{E}-7\ast {\mathrm{MW}}^3\end{array}} $$

The natural logarithm of this average predicted birthweight was used with the observed birthweight to determine a z-score and percentile for each infant.

If an infant fell into the 10th percentile for his or her given characteristics, then they were considered as having FGR. Otherwise they were considered in the pool of controls. Controls were matched to cases using a 2:1 ratio based on study site, maternal age (± 2 years), parity, and infant sex.

### Metabolomic assays

Urine samples were shipped on dry ice and stored at − 80 °C at The Metabolomics Core Lab at Mayo Clinic in Rochester, MN, where their metabolic profiles were analyzed. The samples were prepared and NMR spectra were recorded according to Bruker IVDr (in vitro diagnostics) SOPs. [22] The samples were thawed on ice and mixed with Bruker VERBR urine buffer (phosphate buffer pH 7.4 containing 0.1% TSP-*d*_4_) in 9:1 (*v*/v) ratio. Typically, a 550 μl urine aliquot was transferred to an Eppendorf tube, then 61.1 μl of buffer was added and the mixture was vortexed for 20 s. The sample was spun down at 5000 rpm and 600 μl of supernatant was transferred to 5 mm NMR tubes. Pool samples were prepared by combining 50 μL aliquots of all the samples. The NMR spectra were recorded on a Bruker 600 MHz Avance III HD spectrometer equipped with BBI room temperature probehead and SampleJet auto sampler (Bruker Biospin, Rheinstetten, Germany). The auto sampler temperature was regulated at 6 °C. Tuning, matching, shimming, and pulse calibration were performed in automation mode prior to acquisition. For each sample, two experiments were acquired: 1D noesy with presaturation (noesygppr1d) and homonuclear 2D *J*-resolved (jresgpprqf) at 300 K.^1^H noesy spectral parameters were: p1 ~ 12 μs, ds 4, ns 32, td 64 k, sw 20 ppm (12,019 Hz), aq 2.73 s, d14s, d8 80 ms. The FIDs were multiplied by an exponential weighting function corresponding to a line broadening of 0.3 Hz prior to Fourier transformation. Spectra were automatically referenced and phase and baseline corrected using the Bruker IVDr protocol in the software program Topspin, version 3.5. The 2D *J*-resolved spectral parameters were: F1 domain td 40, sw 0.13 ppm (78 Hz), aq 0.26, F2 domain td 8 k, ds 12, ns 2, sw 16.7 ppm (10,026 Hz), aq 0.41 s, d1 2 s.

After acquisition, the spectra for the four metabolites of interest, were automatically uploaded and analyzed by the Bruker IVDr server. The reports, based on individual samples, and containing concentrations of 18 standard and 8 nonstandard metabolites, were created (Additional file 1). Three of these standard metabolites were the metabolites of interest (acetate, formate, trimethylamine). Tyrosine was not a standard metabolite and was quantified individually in the software program Chenomx NMR, suite 8.2, profiler mode, by fitting experimental spectra to resonant tyrosine peaks at δ 6.88 (d) and 7.18 (d). The concentrations were expressed as mmol/L of urine and mmol/mol of Creatinine (Additional file 2). The quantification of urine metabolites is based on an ERETIC signal generated at 12 ppm. (Additional file 3). The concentrations were normalized to creatinine in the urine due to fluctuations in hydration of the participants.

#### Exploratory metabolite identification

All NMR spectra were uploaded into the software program Chenomx NMR suite 8.2. The spectral region δ 0.5 to 9.4 ppm was divided in 216 custom bins, with region 4.69–4.86 containing residual water resonances excluded. The bin integrals were normalized to total peak area and subsequently used for statistical analysis (Additional file 4). As an alternative approach, we tried excluding the urea region δ 5.3–6.4 and PQN normalization, but it did not improve statistical analysis.

To explore if any other metabolites were associated with FGR, analyses were performed using SIMCA (Soft independent modeling of class analogy) software v14 (MKS Data Analytics Solutions, Umeå, Sweden) for multivariate data analysis. [23] Unsupervised principal component analysis (PCA) was run to detect any innate trends and potential outliers within the data. Supervised partial least squares discriminant analysis (PLS-DA) and orthogonal partial least squares discriminant analysis (OPLS-DA) were performed to obtain additional information on differences in the metabolite composition of groups. PLS-DA and OPLS-DA models were calculated with unit variance scaling, and the results were visualized in the form of score plots to show the group clusters. The VIP (variable importance in the projection) values and regression coefficients were calculated to identify the most important molecular variables for the clustering of specific groups.

### Statistical analysis

Demographic variables (maternal age, maternal BMI, site, parity, gestational age, birthweight and infant gender) were summarized by mean (standard deviation) for continuous variables and N(%) for categorical variables. These characteristics were compared between cases and controls using T-tests and chi-square tests.

#### Four metabolites

The goal of the primary analysis was to determine if there exists an association between the four metabolite concentrations at either trimester with odds of FGR. This included multivariable conditional logistic regression for each of the four metabolites to determine. Demographic variables that were not used in calculating the outcome (maternal age, BMI, and parity) were considered for inclusion in the model along with metabolite concentration at each trimester. Interquartile odds ratios (IORs) with 95% confidence intervals (CIs) were calculated for FGR to produce estimates of ORs customized to a unit of change reflecting the difference between the 75th percentile and 25th percentile of each metabolite concentration*.*

#### Exploratory metabolites

The NMR spectra were custom binned into potential metabolite representatives so that each patient had one measure of “proportion of spectrum” in the first trimester and one in the second trimester for each potential metabolite. Median concentrations were estimated within each bin for each trimester and the difference in medians between cases and controls was calculated. For each trimester, the absolute differences were ranked in descending order and the 25 biggest were noted. For these 50 bins, within each bin and for each trimester, a nonparametric Wilcoxon rank sum test was performed to compare median “proportion of spectrum” between cases and controls. The Benjamini-Hochberg method was used to adjust for multiple testing. Results were then listed in order of ascending *p*-value and used to consider which metabolites may merit further exploration regarding an association with FGR.

*P*-values less than 0.05 were considered statistically significant and all analyses were conducted using SAS version 9.4 (Cary, NC).

The STROBE (Strengthening the Reporting of Observational Studies in Epidemiology) guidelines were used to ensure the reporting of this observational study. [24]

## Results

The 53 cases were matched 2:1 to 106 controls for a total of 159 subjects resulting in a total of 318 urine samples. Of the original 159 patients, 158 had sufficient data to be included in the analysis (one subject had unreadable first trimester spectroscopy analysis results).

Demographic comparison results, as shown in Table 1, were as expected given the construction of the FGR formula and the matching on maternal age, site, parity, and infant gender. There was no difference in maternal or infant characteristics with the exception of infant gestational age and birthweight. Maternal BMI was similar between cases and controls. Cases had a statistically significantly lower gestational age at birth (38.6 vs 39.8, *p* < 0.001), and lower birthweight (2527 g vs 3471 g, *p* < 0.001).

**Table 1:** Demographic data [mean(SD)]

Variable		Overall (*n* = 158)	FGR Case (*n* = 53)	Control (*n* = 105)	*p*-value*
Maternal Age	(yrs)	29.4 (6.2)	29.5 (6.4)	29.3 (6.1)	0.91
Maternal BMI	(kg/m^2^)	27.9 (7.0)	27.9 (7.6)	27.8 (6.7)	0.98
Site					1.0
	UCSF	29	10 (18%)	19 (19%)	
	UMN	21	7 (13%)	14 (13%)	
	URMC	93	31 (59%)	62 (58%)	
	UW	15	5 (10%)	10 (9%)	
Parity					0.86
	0	94	31 (58%)	63 (60%)	
	≥1	64	22 (42%)	42 (40%)	
Gestational Age at Birth	(wks)	39.4 (1.8)	38.6 (2.4)	39.8 (1.1)	< 0.0001
Birth weight	(g)	3154 .3 (621)	2526.5 (421)	3471.1 (448)	< 0.0001
Sex					0.96
	Male	75	25 (47%)	50 (48%)	
	Female	83	28 (53%)	55 (52%)	
Smoker at T1		14 (9%)	5 (10%)	9 (9%)	1.00
Cigarettes per wk. (*n* = 14)	Med (IQR)	21 (3–35)	28 (25–35)	7 (2–21)	0.14
Pregestational Diabetes		2	0	2 (2%)	0.55
Chronic Hypertension		4	2 (4%)	2 (2%)	0.60

*p-values for Maternal Age, BMI, Gestational Age, and Birthweight are from T-tests. *P*-values Site, Parity, Sex, and Smoker are from chi-square tests. *P*-value for Cigarettes per week is from Wilcoxon rank sum test. *P*-values for Pregestational Diabetes and Hypertension are from Fisher’s exact test

### Four metabolites

The median concentrations of the four metabolites are listed in Table 2. None of the demographic variables of interest differed significantly between cases and controls, and were not included in the model. The final model consisted of only the main effects of the metabolite levels in each trimester. The resulting estimates of Interquartile ORs for each metabolite are displayed in Table 3; any association between each of the four metabolites and odds of FGR was found to be non-significant.

**Table 2:** Median [IQR] mmol/mol creatinine of each metabolite by trimester

Variable	Trimester	Overall	FGR Case	Control
Acetic Acid	1	11 [7–17]	10 [6–16]	11 [8–18]
	2	12 [7–17]	12 [7–19]	11 [7–17]
Trimethylamine	1	4 [3–6]	4 [3–5]	4 [3–6]
	2	4 [3–5]	3 [3–5]	4 [3–5]
Formic Acid	1	19 [13–29]	19 [12–31]	19 [14–28]
	2	25 [18–36]	25 [18–37]	25 [18–35]
Tyrosine^a^	1	17 [13–23]	16 [8–22]	18 [13–24]
	2	18 [13–25]	19 [12–24]	18 [13–25]

^a^Some subjects were missing tyrosine spectroscopy analysis results, thus *n* = 153 subjects were used in analysis of tyrosine

**Table 3:** Interquartile Odds Ratios of FGR for each metabolite

Variable	Trimester	Odds Ratio	95% CI		*p*-value
			Min	Max	
Acetic Acid	1	0.93	0.77	1.11	0.42
	2	1.12	0.86	1.46	0.40
Trimethylamine	1	0.90	0.59	1.35	0.60
	2	1.20	0.80	1.79	0.38
Formic Acid	1	1.07	0.64	1.79	0.78
	2	1.06	0.74	1.53	0.73
Tyrosine^a^	1	0.74	0.49	1.10	0.14
	2	1.16	0.76	1.78	0.50

Interquartile ranges for acetic acid: 10, trimethylamine: 2.5, formic acid: 17, and tyrosine: 11

^a^Some subjects were missing tyrosine spectroscopy analysis results, thus *n* = 153 subjects were used in analysis of tyrosine

### Exploratory metabolites

**Fig. 1:**
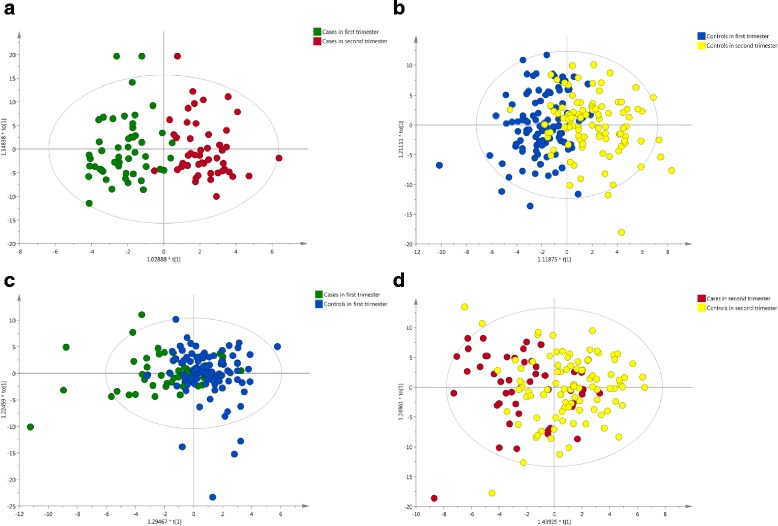
**a**-**d**: OPLS-DA score plots in the predictive (x-axis) and orthogonal (y-axis) components of ^1^H NMR spectral data of pregnant women urine samples

Metabolite identification (as described under Methods) was effective when comparing first trimester case samples to second trimester case samples and first trimester control samples to second trimester control samples, as in Fig. 1, a and b; however, when we compared case samples versus control samples within a trimester, good separation could not be visualized; see Fig. 1, c and d.

The custom binning of the NMR spectroscopy resulted in 215 bins expected to represent separate metabolites. The 25 largest differences in relative concentration between cases and controls in the first trimester ranged from 0.000256 to 0.006265. The 25 largest differences in relative concentration between cases and controls in the second trimester ranged from 0.000347 to 0.004184. After adjusting for multiple testing, none of the *p*-values were found to be significant. The bins that correlated with the largest differences in the first trimester were attributed to 1-Methylnicotinamide, Lysine, Proline betaine, 3-Hydroxybutyrate/3-Aminoisobutyrate, Creatinine and Guanidoacetate. The bins correlated with the largest differences in the second trimester were attributed to Xanthyrenate, Histidine, Acetate + N-Phenylacetyl glutamine, Urea and Carnitine. Again, none of these were of significance.

## Discussion

We found no significant association between the relative concentration level of each of the four metabolites of interest and odds of FGR. In addition, results did not reveal any significant differences in the exploratory urinary metabolic profiles. As expected, compared with controls, cases delivered earlier (38.6 vs 39.8, *p* < 0.001), and had lower birthweights (2527 g vs 3471 g, *p* < 0.001).

Pregestational diabetes and chronic hypertension, other noted factors that can contribute to fetal growth restriction, were rare in this cohort (pre-gestational diabetes: 0/53 cases, 2/109 controls, chronic hypertension: 2/53 cases, 2/109 controls) and thus were not included in our analyses. Smoking among subjects was equally proportionate among cases and controls.

The strengths of our study include the large cohort of patients in the TIDES group (758 patients total enrolled), which allowed the relatively rare nature of fetal growth restriction to yield a large number of cases (53) with matched controls. The patients came from 4 different geographic areas of the country and this contributed to the diversity of the group. Additionally, our analysis involved conditional logistic regression which helps account for the bias inherent to a case-control study. Another strength was the emphasis on the accuracy of our data and our sample runs in the NMR spectroscopy lab at Mayo. Trial runs were performed on mock urine samples before initiating processing and NMR spectroscopy on our actual samples. This limited human error as well as machine error. Finally, given the sensitive nature of NMR spectroscopy, it is vital to assemble a team with members from the metabolomics lab as well as the clinical side to ensure data collection and results are valid and not the result of sample processing error, lab mishandling, software error or assumptions made on poor understanding of the spectra profiles. The collaborative nature of this project with expert spectroscopists as well as clinicians allowed for accurate collection and analysis of data while at the same time maintaining a focus on the potential clinical significance of our findings.

NMR spectroscopy did confirm a significant metabolic shift between first and second trimesters through multivariate projection analysis such as principal component analysis (PCA) and orthogonal-partial least squares discriminatory analysis (OPLS-DA); however, the accessibility of inexpensive methods (eg. ultrasound) to determine gestational age undermines the clinical usefulness of NMR spectroscopy to discern what trimester a pregnant patient is in. Still, this result does prove the technology reveals that the metabolites are undergoing a significant change. In another recent study, investigators found that a 50% increase in each of three separate third trimester urine metabolites is correlated with an increase in birth weight of 1%–2.4% (5-11 g). [25] Though these results may have statistical significance in that study, and might warrant further investigation, they too have little clinical value at this time, considering the small size of the increase.

The current results do not to show the metabolomics to offer potential biomarkers of FGR but do offer intriguing preliminary data to pursue further given the potential of metabolomics to yield clinically useful and individualized results from a low risk, non-interventional sample early in pregnancy. Further studies are warranted not only using NMR spectroscopy, but also applying highly sensitive tandem mass spectrometry based approaches utilizing timely collection of plasma samples. Though the gathering of non-interventional samples such as urine is very low cost, the running of urine samples by NMR spectroscopy is very expensive; plasma based measurements applying sensitive and less expensive mass spectrometry based metabolomics approaches may be considered in the future to assist diagnosis of conditions in obstetrics.

The primary limitation to our study is the exploratory nature of this area. There is very little metabolomics literature available on pregnant patients. Conversely, this might also be considered the greatest strength; when a path is new, every step forward has a greater impact on the direction traveled. Also, the retrospective nature of our study was limiting to the extent that we could not control what data had already been collected. It would have been useful to have chosen a stricter definition of FGR (5th percentile or 3rd percentile). With a tighter definition, our specificity would increase, but at the expense of sensitivity. Given the exploratory nature of this work to assess if urinary metabolites are a plausible means by which to screen for FGR, we chose a higher cut off to achieve a data set that was interpretable. A sensitivity analysis was conducted with cases in the 5th percentile and we found similar results. Additionally, because it is not an ongoing study, we cannot add to our data to incorporate further subjects in future. It may have been useful to include Doppler information from ultrasounds with the focus on looking for signs of growth restriction had we been given the choice. This is the nature of studying rare outcomes with a lack of time and funding necessary for a prospective study. Our NMR spectroscopy data will be added to the Bruker company’s database of labs using their machines.

## Conclusions

In conclusion, we did not detect changed odds of FGR based on the concentration of urinary metabolites (acetate, formate, trimethylamine and tyrosine) in either trimester. Additionally, we did not detect differences in the first and second trimester urinary metabolic profiles of pregnant patients with FGR compared to controls. This contradicts prior work, and highlights the need for more rigorous investigation and data collection in this area before metabolomics can be clinically applied to obstetrics.

## Additional files

Additional file 1: Bruker Analysis Report. Analysis report generated from Bruker software containing concentrations of 18 standard and 8 nonstandard metabolites for all samples. (PDF 99 kb)

Additional file 2: Tyrosine concentrations for all samples. Tyrosine was not a standard metabolite and was quantified individually in the software program Chenomx NMR, suite 8.2, profiler mode, by fitting experimental spectra to resonant tyrosine peaks at δ 6.88 (d) and 7.18 (d). The concentrations were expressed as mmol/L of urine and mmol/mol of Creatinine. (XLSX 135 kb)

Additional file 3: ^1^H NMR Bruker IVDr spectrum of pooled urine sample. The quantification of urine metabolites is based on an ERETIC signal generated at 12 ppm. (PPTX 350 kb)

Additional file 4: Binned spectrum line using total area normalization. All NMR spectra were uploaded into the software program Chenomx NMR suite 8.2. The spectral region δ 0.5 to 9.4 ppm was divided in 216 custom bins, with region 4.69–4.86 containing residual water resonances excluded. The bin integrals were normalized to total peak area and subsequently used for statistical analysis. (XLSX 703 kb)

## Abbreviations

ACOG: The American College of Obstetricians and Gynecologists
CI: Confidence interval
FGR: Fetal growth restriction
IOR: Interquartile odds ratio
IVD: In vitro diagnostics
OPLS-DA: Orthogonal partial least squares discriminant analysis
PCA: Principal component analysis
PLS-DA: Partial least squares discriminant analysis
SD: Standard deviation
SGA: Small for gestational age
SIMCA: Soft Independent Modeling of Class Analogy
STROBE: Strengthening the Reporting of Observational Studies in Epidemiology
TIDES: The Infant Development and the Environment Study
VIP: Variable importance in the projection
