# Supplementary material for: First and second trimester urinary metabolic profiles and fetal growth restriction: an exploratory nested case-control study within the infant development and environment study
Source: BMC Pregnancy Childbirth. 2018 Feb 8;18:48. doi: 10.1186/s12884-018-1674-8 (PMC5806311; doi:10.1186/s12884-018-1674-8)
Supplement: Supplementary file 1 — Bruker Analysis Report. Analysis report generated from Bruker software containing concentrations of 18 standard and 8 nonstandard metabolites for all samples. (PDF 99 kb) [file 12884_2018_1674_MOESM1_ESM.pdf]

Analysis Report Urine-Screening

Sample: 5_pool1.100000.10r

Measuring Date: 01-Apr-2016 03:53:05
Reporting Date: 01-Apr-2016 11:02:26
Analysis Version: 0.3
Number of Pages: 3

Sample Receiving Date: ______________________

Comment: ______________________

Disclaimer

RESEARCH USE ONLY: This is no clinical diagnostic analysis report. Must not be used for clinical (medical or IVD) diagnosis or for patient management!

General Remarks

A list of 26 metabolites is quantified automatically from the spectrosopic data. Metabolite concentrations given in mmol/L and mmol/mol creatinine are compiled in the second and third column, respectively.
The limits of detection (LOD) are not yet available and replaced with the labeling 'N.A.'. LODs will be provided in future releases.

Compound	Concentration		LOD
	mmol/L	mmol/mol Crea	mmol/mol Crea

Geschäftsführer: Dr. Falko Busse | Dr. Iris Mangelschots | Stephan Westermann

Silberstreifen 4
76287 Rheinstetten, Germany
Tel. +49(0)721 5161-0
Fax. +49(0)721 517101
nmr@bruker-biospin.com
www.bruker-biospin.com

Deutsche Bank AG Karlsruhe
Konto 13 15 16 · BLZ 660 700 04
IBAN: DE87 6607 0004 0013 1516 00
SWIFT-BIC: DEUTDESM660

USt-Ident.-Nr DE 143 239 759
WEEE-Reg.-Nr. DE 43 181 702
Steuer-Nr. 31190/39205
Handelsregister Mannheim HRB 10 23 68
Sitz der Gesellschaft: 76287 Rheinstetten

Bruker BioSpin GmbH

Targeted Analysis (Quantification)

Standard Compounds

Compound	Concentration[1] mmol/L	mmol/mol Crea	LOD mmol/mol Crea
Creatinine	8.2		
Creatine	0.97	119	N.A.
D-Glucose-beta	0.24	29	N.A.
Alanine	0.46	56	N.A.
Lactic Acid	0.71	87	N.A.
Acetic Acid	0.10	12	N.A.
Succinic Acid	0.17	21	N.A.
Citric Acid	3.51	428	N.A.
Dimethylamine	0.24	29	N.A.
Trimethylamine	0.03	3	N.A.
Betaine	0.17	21	N.A.
Glycine	2.56	313	N.A.
Fumaric Acid	0.01	1	N.A.
Formic Acid	0.17	20	N.A.
1-Methylnicotinamide	0.11	13	N.A.
N,N-Dimethylglycine	0.04	5	N.A.
Trigonelline	0.09	11	N.A.
Hippuric Acid	1.57	191	N.A.

[1] Please take notice of the general remarks and the annotations mentioned on the cover page.

Non-Standard Compounds

Compound	Concentration[1]		LOD
	mmol/L	mmol/mol Crea	mmol/mol Crea
D-Lactose	0.45	55	N.A.
Paracetamol	0.01	1	N.A.
Paracetamol-glucuronide	0.00	0	N.A.
Acetone	0.05	6	N.A.
3-Hydroxybutyric Acid	0.00	0	N.A.
Acetoacetic Acid	0.18	22	N.A.
Benzoic Acid	0.03	3	N.A.
Ethanol	0.00	0	N.A.

[1] Please take notice of the general remarks and the annotations mentioned on the cover page.
