# Supplementary material for: First and second trimester urinary metabolic profiles and fetal growth restriction: an exploratory nested case-control study within the infant development and environment study
Source: BMC Pregnancy Childbirth. 2018 Feb 8;18:48. doi: 10.1186/s12884-018-1674-8 (PMC5806311; doi:10.1186/s12884-018-1674-8)
Supplement: Supplementary file 3 — 1H NMR Bruker IVDr spectrum of pooled urine sample. The quantification of urine metabolites is based on an ERETIC signal generated at 12 ppm. (PPTX 350 kb) [file 12884_2018_1674_MOESM3_ESM.pptx]

## Slide 1
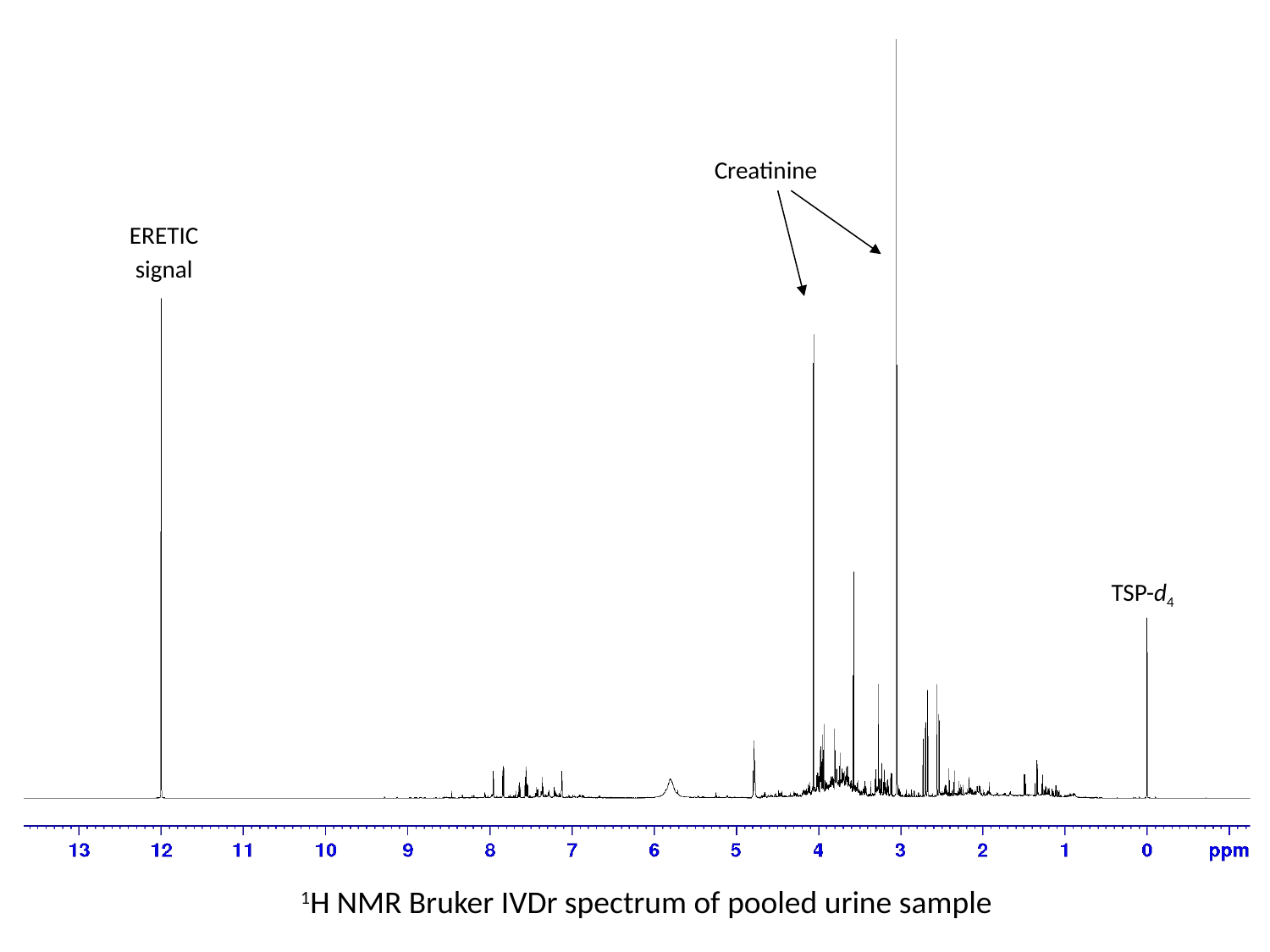

Creatinine
ERETIC signal
TSP-d4
1H NMR Bruker IVDr spectrum of pooled urine sample

## Slide 2
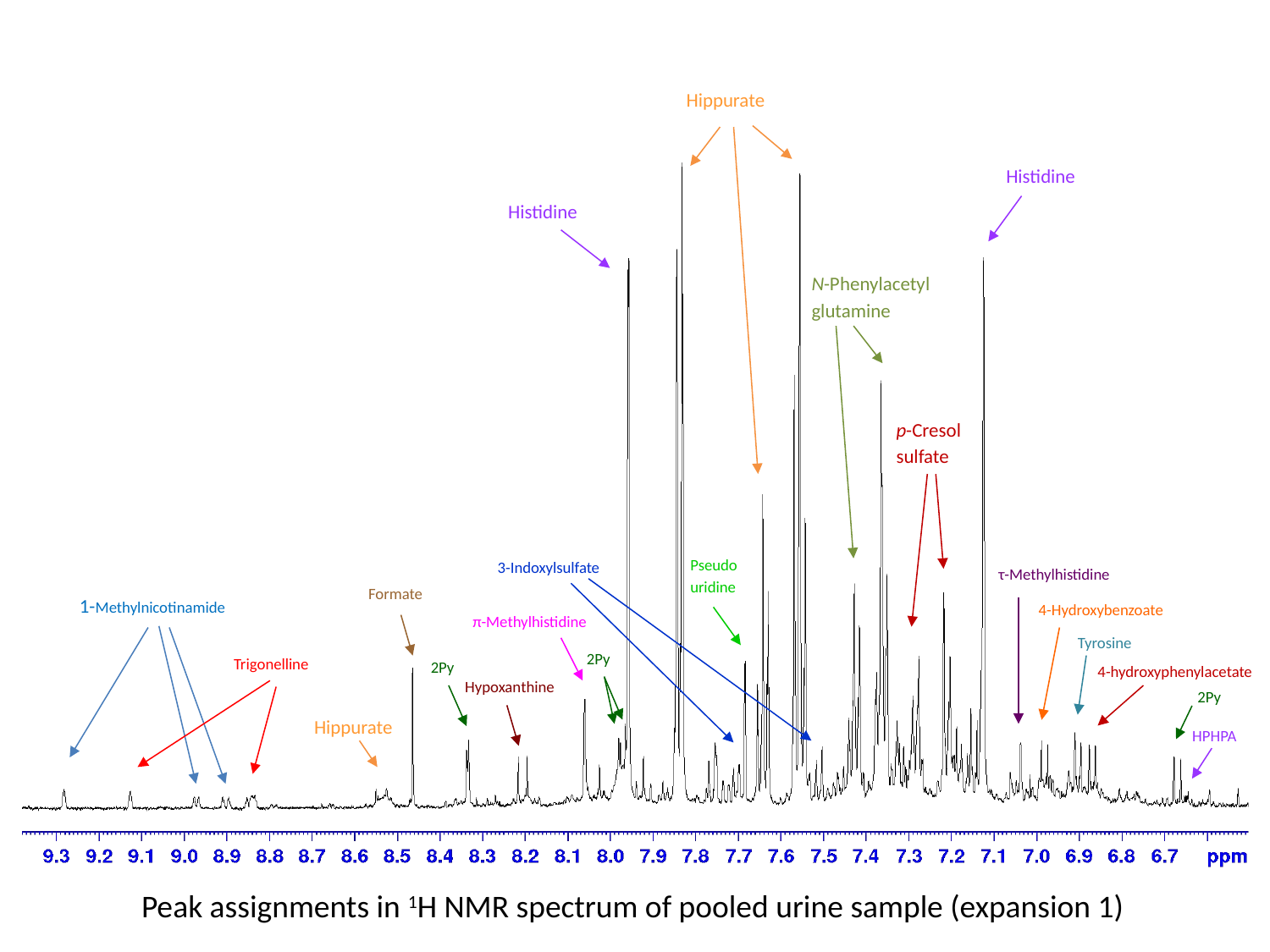

Hippurate
Histidine
Histidine
N-Phenylacetyl glutamine
p-Cresol sulfate
Pseudouridine
3-Indoxylsulfate
τ-Methylhistidine
Formate
1-Methylnicotinamide
4-Hydroxybenzoate
π-Methylhistidine
Tyrosine
2Py
Trigonelline
2Py
4-hydroxyphenylacetate
Hypoxanthine
2Py
Hippurate
HPHPA
Peak assignments in 1H NMR spectrum of pooled urine sample (expansion 1)

## Slide 3
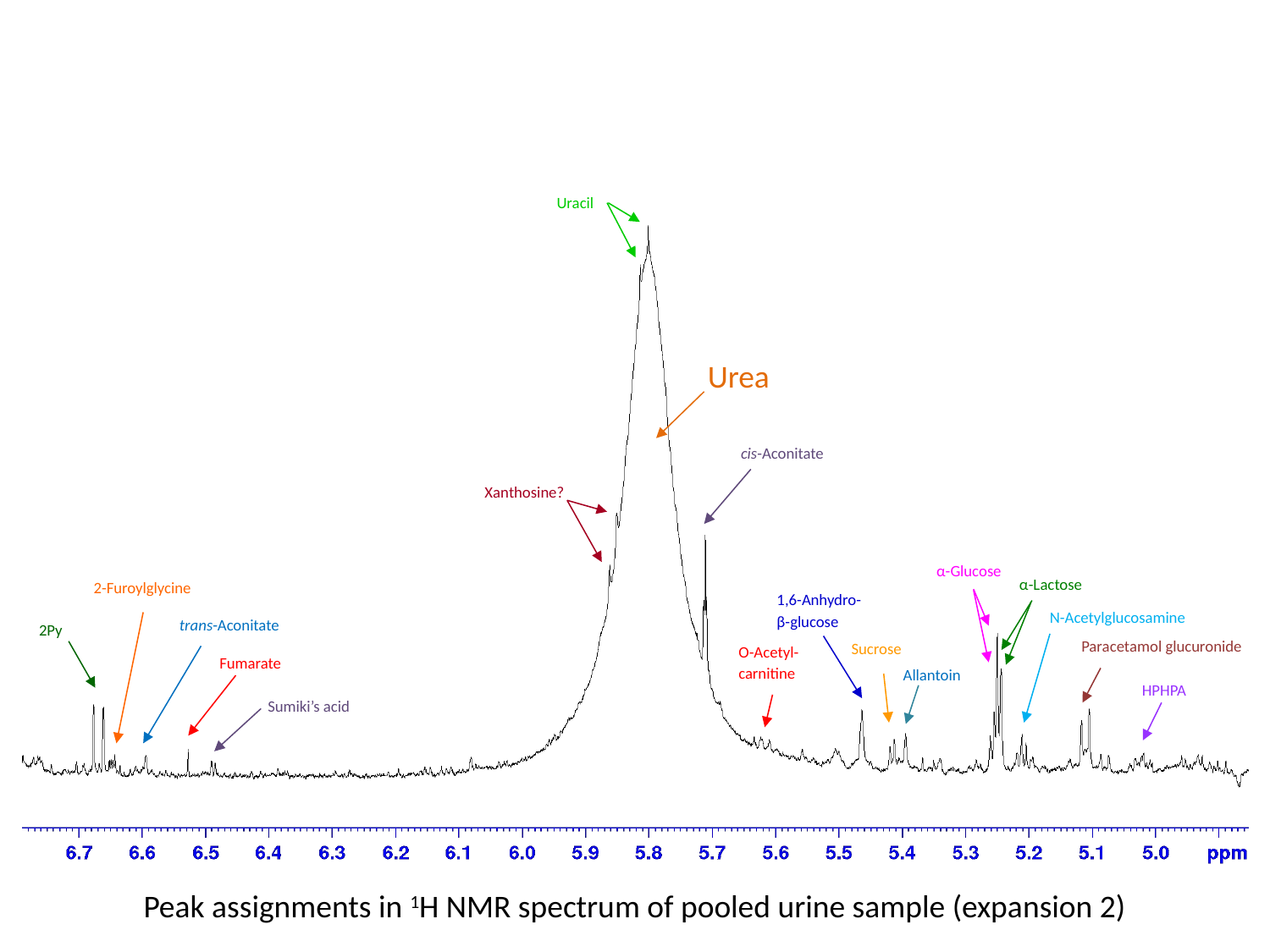

Uracil
Urea
cis-Aconitate
Xanthosine?
α-Glucose
α-Lactose
2-Furoylglycine
1,6-Anhydro- β-glucose
N-Acetylglucosamine
trans-Aconitate
2Py
Paracetamol glucuronide
Sucrose
O-Acetyl-carnitine
Fumarate
Allantoin
HPHPA
Sumiki’s acid
Peak assignments in 1H NMR spectrum of pooled urine sample (expansion 2)

## Slide 4
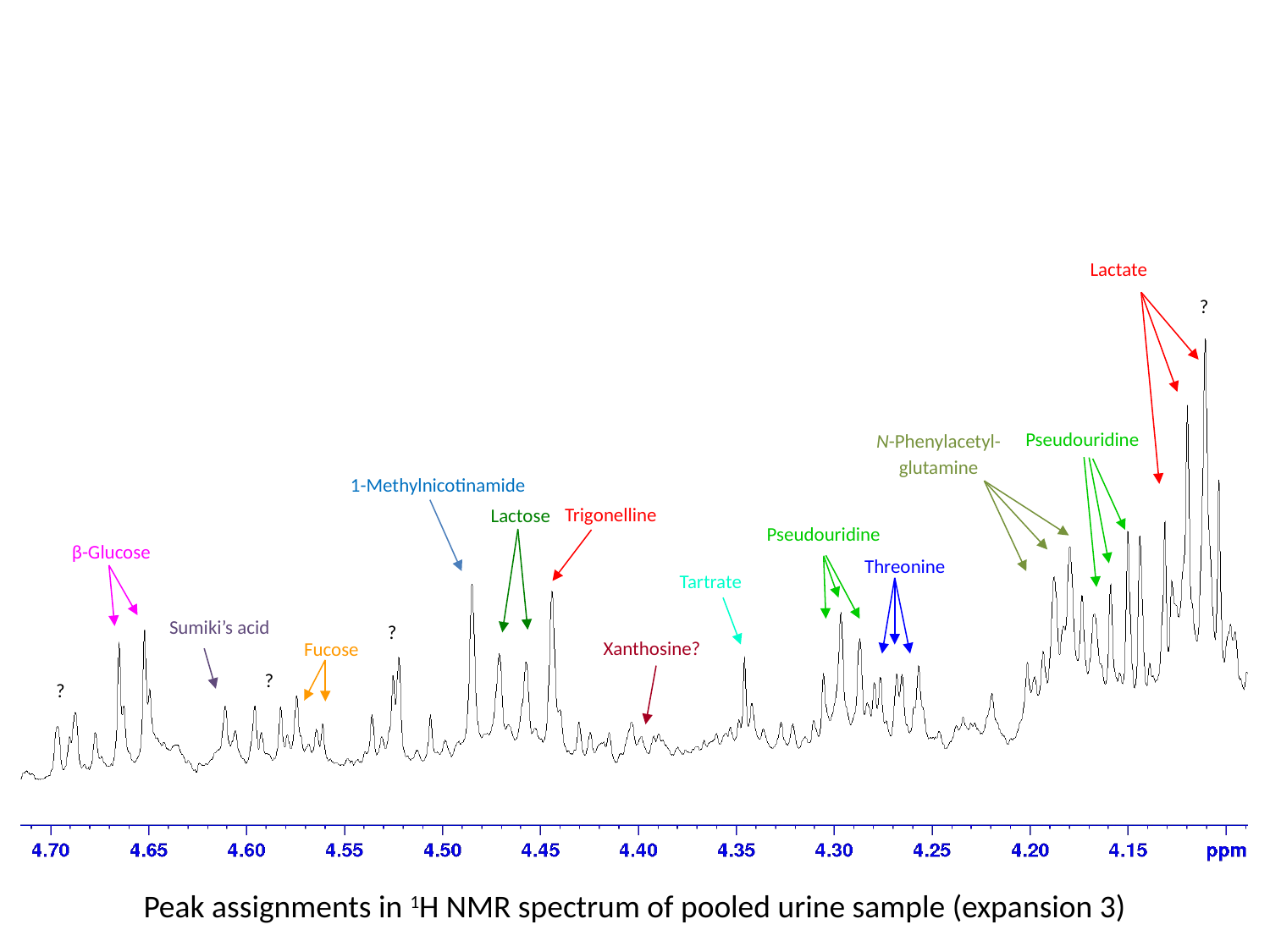

Lactate
?
Pseudouridine
N-Phenylacetyl-glutamine
1-Methylnicotinamide
Trigonelline
Lactose
Pseudouridine
β-Glucose
Threonine
Tartrate
Sumiki’s acid
?
Xanthosine?
Fucose
?
?
Peak assignments in 1H NMR spectrum of pooled urine sample (expansion 3)

## Slide 5
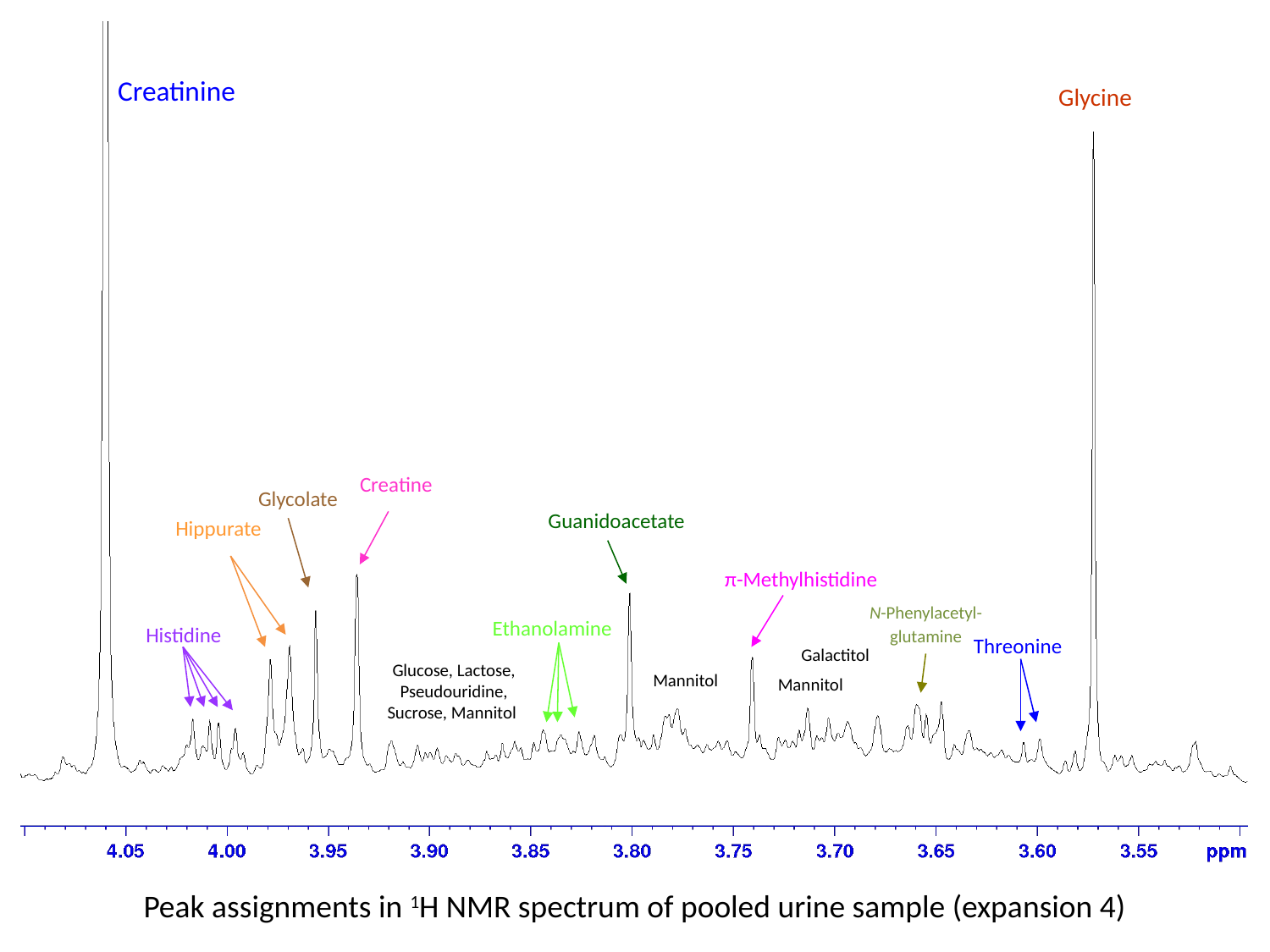

Creatinine
Glycine
Creatine
Glycolate
Guanidoacetate
Hippurate
π-Methylhistidine
N-Phenylacetyl-glutamine
Ethanolamine
Histidine
Threonine
Galactitol
Glucose, Lactose,
Pseudouridine,
Sucrose, Mannitol
Mannitol
Mannitol
Peak assignments in 1H NMR spectrum of pooled urine sample (expansion 4)

## Slide 6
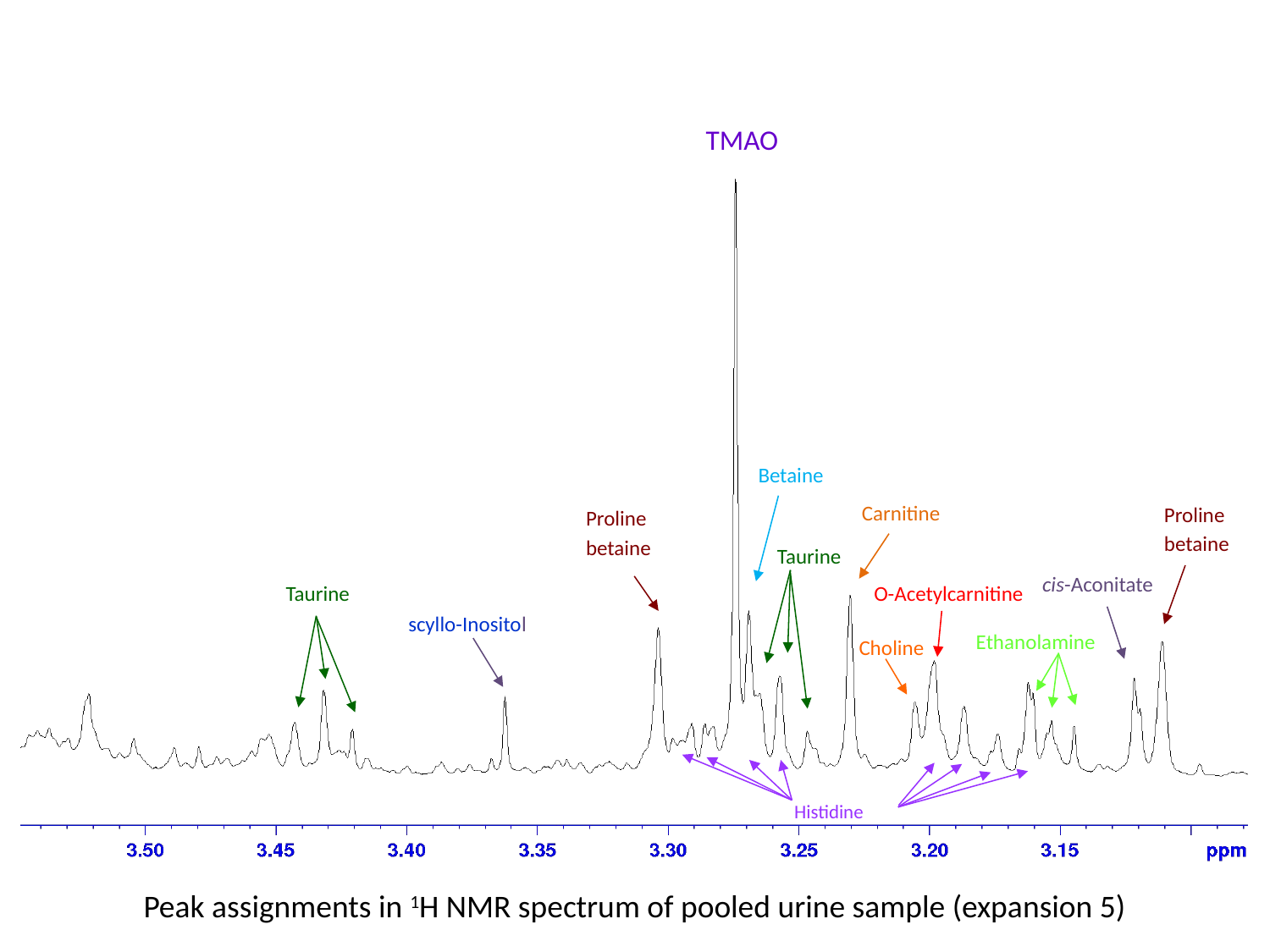

TMAO
Betaine
Carnitine
Proline betaine
Proline betaine
Taurine
cis-Aconitate
Taurine
O-Acetylcarnitine
scyllo-Inositol
Ethanolamine
Choline
Histidine
Peak assignments in 1H NMR spectrum of pooled urine sample (expansion 5)

## Slide 7
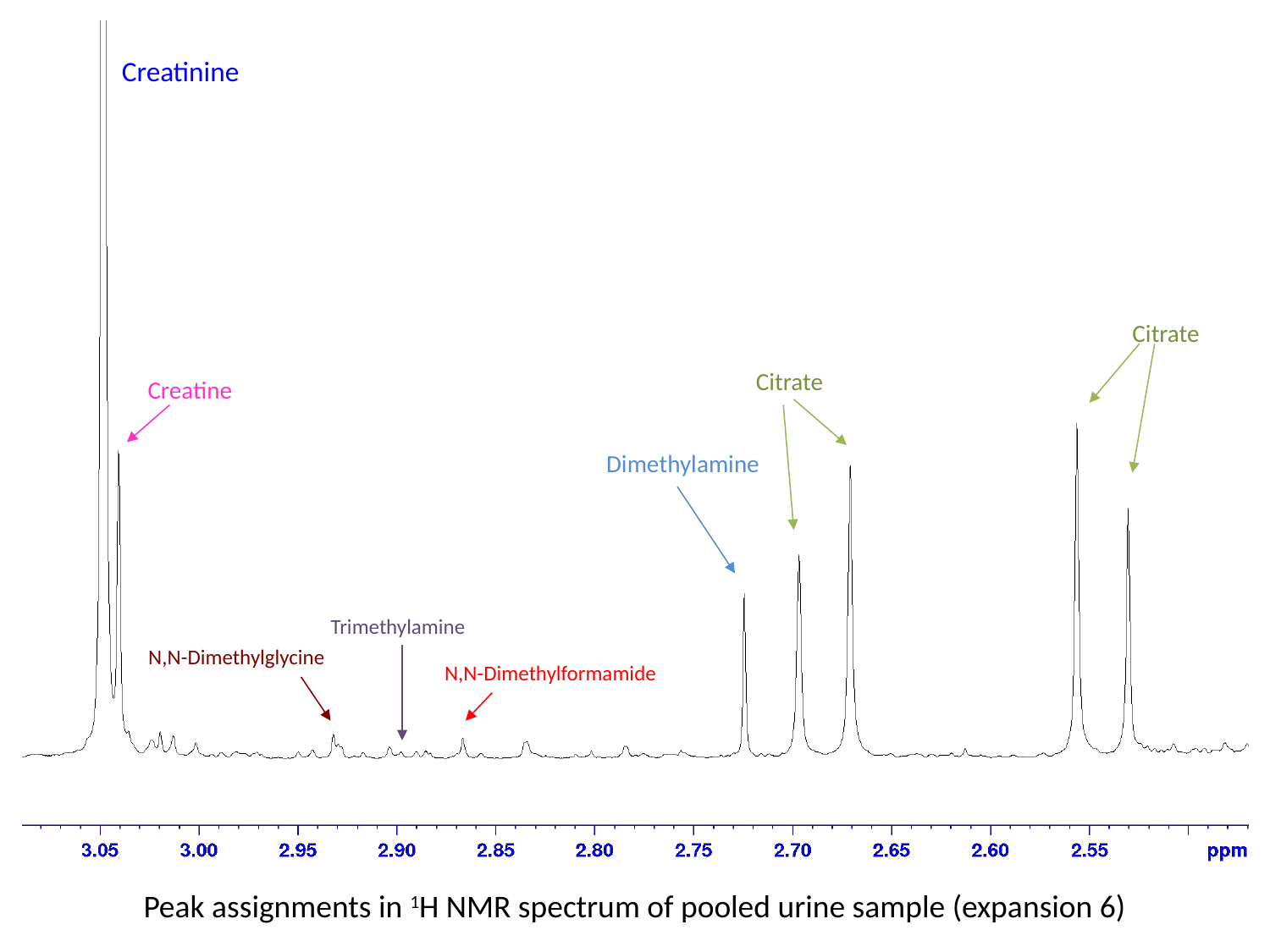

Creatinine
Citrate
Citrate
Creatine
Dimethylamine
Trimethylamine
N,N-Dimethylglycine
N,N-Dimethylformamide
Peak assignments in 1H NMR spectrum of pooled urine sample (expansion 6)

## Slide 8
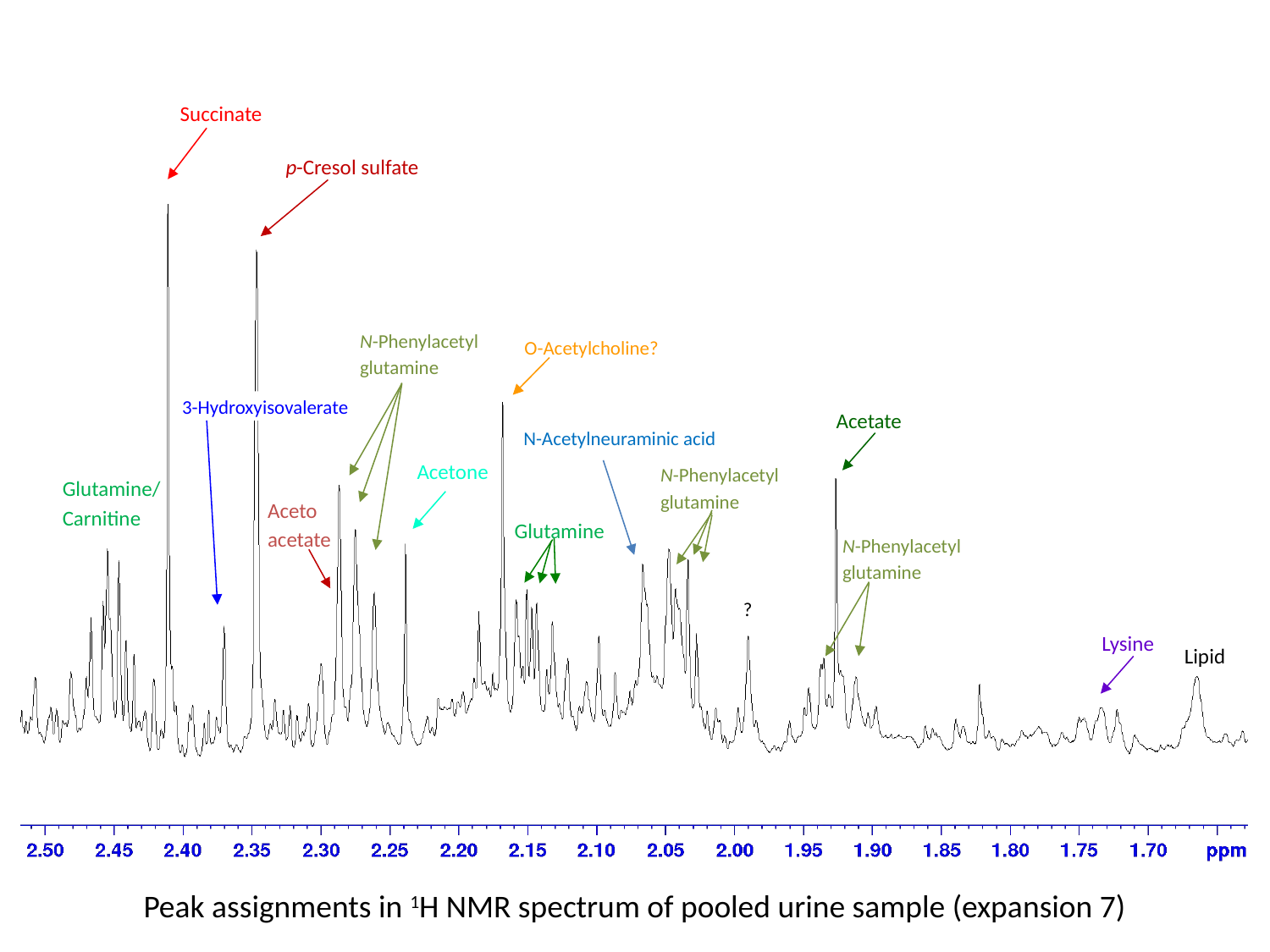

Succinate
p-Cresol sulfate
N-Phenylacetyl glutamine
O-Acetylcholine?
3-Hydroxyisovalerate
Acetate
N-Acetylneuraminic acid
Acetone
N-Phenylacetyl glutamine
Glutamine/Carnitine
Aceto acetate
Glutamine
N-Phenylacetyl glutamine
?
Lysine
Lipid
Peak assignments in 1H NMR spectrum of pooled urine sample (expansion 7)

## Slide 9
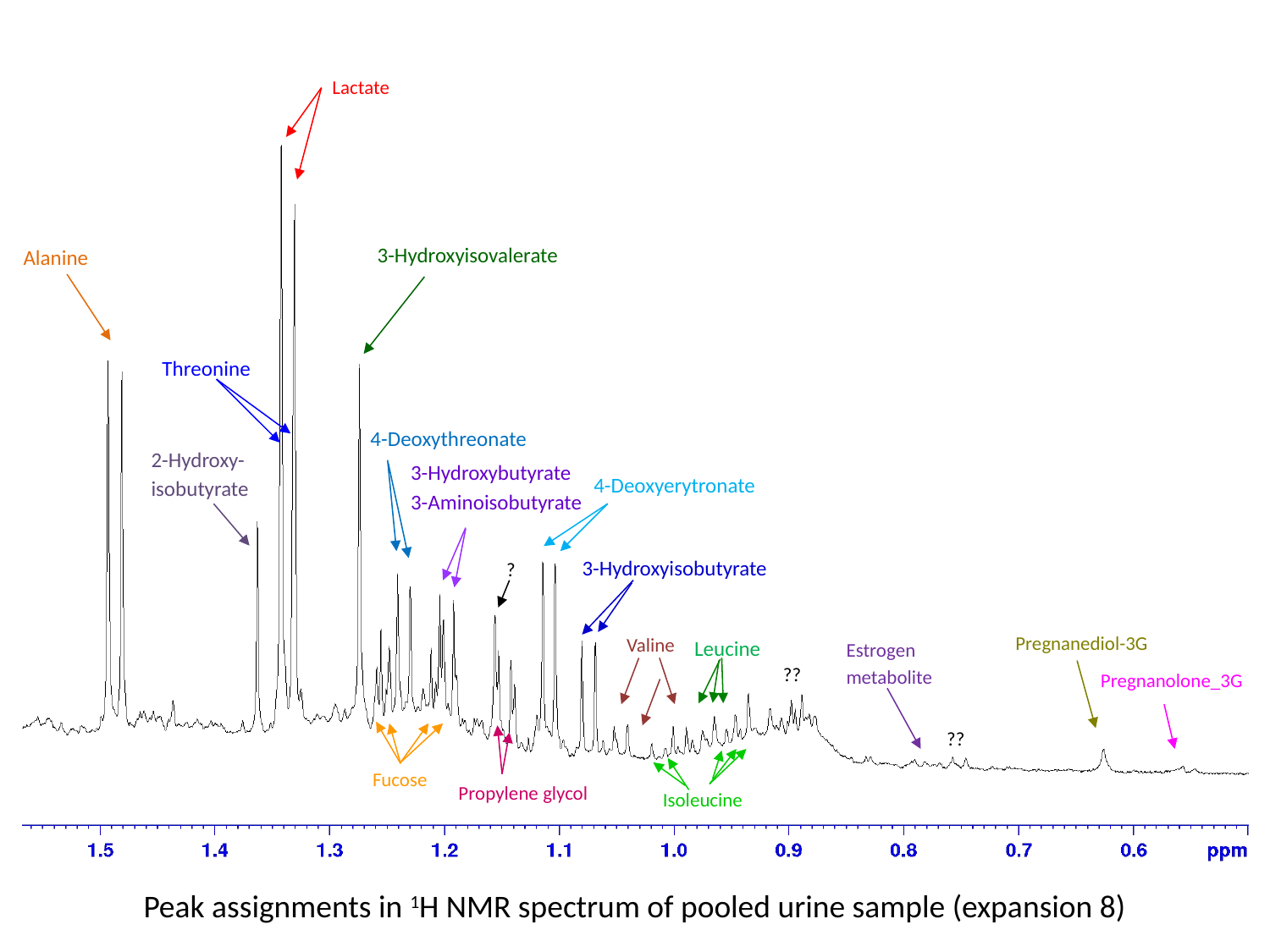

Lactate
3-Hydroxyisovalerate
Alanine
Threonine
4-Deoxythreonate
2-Hydroxy- isobutyrate
3-Hydroxybutyrate 3-Aminoisobutyrate
4-Deoxyerytronate
3-Hydroxyisobutyrate
?
Pregnanediol-3G
Valine
Leucine
Estrogen metabolite
??
Pregnanolone_3G
??
Fucose
Propylene glycol
Isoleucine
Peak assignments in 1H NMR spectrum of pooled urine sample (expansion 8)
